# The association between educational status and colorectal neoplasia: results from a screening cohort

**DOI:** 10.1007/s00384-023-04383-z

**Published:** 2023-04-05

**Authors:** Sarah Wernly, Georg Semmler, Dagmar Schaffler-Schaden, Maria Flamm, Elmar Aigner, Christian Datz, Bernhard Wernly

**Affiliations:** 1 Department of Internal Medicine, General Hospital Oberndorf, Teaching Hospital of the Paracelsus Medical University, Salzburg, Austria; 2 Division of Gastroenterology and Hepatology, Department of Medicine III, Medical University of Vienna, Vienna, Austria; 3 Institute of General Practice, Family Medicine and Preventive Medicine, Paracelsus Medical University, Salzburg, Austria; 4 Clinic I for Internal Medicine, University Hospital Salzburg, Paracelsus Medical University, Salzburg, Austria

**Keywords:** Educational status, Colorectal neoplasia, Colorectal cancer, Public health

## Abstract

**Introduction:**

Educational status is used as a proxy for socioeconomic status. While lower levels of education are generally associated with poorer health, the data on the relationship between educational status and colorectal neoplasia is heterogenous. The aim of our study was to examine this relationship and to adjust the association between educational status and colorectal neoplasia for other health parameters.

**Methods:**

We included 5977 participants undergoing a screening colonoscopy in Austria. We split the cohort into patients with lower (*n* = 2156), medium (*n* = 2933), and higher (*n* = 459) educational status. Multivariable multilevel logistic regression models were fitted to evaluate the association between educational status and the occurrence of any or advanced colorectal neoplasia. We adjusted for age, sex, metabolic syndrome, family history, physical activity, alcohol consumption, and smoking status.

**Results:**

We found that the rates of any neoplasia (32%) were similar between the educational strata. However, patients with higher (10%) educational status evidenced significantly higher rates of advanced colorectal neoplasia compared to medium (8%) and lower (7%) education. This association remained statistically significant after multivariable adjustment. The difference was entirely driven by neoplasia in the proximal colon.

**Conclusion:**

Our study found that higher educational status was associated with a higher prevalence of advanced colorectal neoplasia compared to medium and lower educational status. This finding remained significant even after adjusting for other health parameters. Further research is needed to understand the underlying reasons for the observed difference, especially with regard to the specific anatomical distribution of the observed difference.

## Introduction

Colorectal cancer (CRC) is among the most common cancers worldwide and causes significant morbidity and mortality [1]. The development of colorectal carcinomas from colorectal neoplasia (adenoma or sessile serrated lesions) over a period of several years makes screening for CRC possible through non-invasive and invasive methods, including endoscopy [2]. Endoscopy is a safe method for removing precancerous growths and has been shown to reduce the incidence of CRC and CRC-related mortality [3–5]. Participation in CRC screening, cancer stage at diagnosis, and CRC-related mortality have all been linked to socioeconomic status [6, 7]. The relationship between socioeconomic status and health outcomes is complex and not fully understood. While lower levels of socioeconomic status are generally associated with worse health outcomes, some studies have found that higher levels of socioeconomic status are associated with increased risk for certain conditions, including colorectal neoplasia and CRC [8]. This unexpected association between education and colorectal neoplasia is not fully understood and may be influenced by other factors such as obesity, physical activity, and cardiovascular risk [9]. It is unclear whether the relationship between socio-economic status and colorectal health is independent of this differential distribution of cardiometabolic and carcinogenic risk factors.

Education is often used as a proxy for socioeconomic status, and lower levels of education have been linked to higher rates of morbidity and mortality [10]. The International Standard Classification of Education (ISCED) is a well-established method for categorizing educational status [11]. In this study, we used the newest version of the ISCED to evaluate the association between educational status and colorectal neoplasia prevalence in a Central European screening cohort [11]. The potential associations between colorectal neoplasia and educational status might have implications for both public health and individual patient counseling. From a public health standpoint, the results of this study could contribute to our understanding of the impact of socioeconomic status on health outcomes. From a clinical perspective, the results of this study may be useful in counseling individual patients.

The aim of this study is to evaluate the distribution of colorectal neoplasia in a central European screening cohort based on educational status, using the ISCED to categorize educational status. The study also evaluates the association between educational status and colorectal neoplasia prevalence independent of known risk factors such as age, sex, and cardiometabolic risk factors.

## Methods

### Subjects

Subjects in this study were taken from the Salzburg Colon Cancer Prevention Initiative (Sakkopi), a cohort of asymptomatic individuals who underwent screening for colorectal cancer at a single center in Austria between January 2007 and March 2020. The total sample consisted of 5977 participants. Clinical and laboratory data were collected for all subjects. [12, 13]. Also, patients completed a questionnaire about their medical history. We defined and calculated body mass index (BMI), arterial hypertension, smoking status, dyslipidemia, and metabolic syndrome according to current guidelines [14, 15]. We categorized the participants’ education level into three categories — lower, medium, and higher education — based on a recent publication by Schneider [11]. Specifically, we categorized patients with GISCED 1 and 2 into low, 3 and 4 into medium, and 5 and 6 into high education.

### Statistical analysis

Continuous data were analyzed using the Mann–Whitney *U* test or Student’s *T* test, depending on the distribution of the data, and are presented as median ± interquartile range (IQR) or mean ± standard deviation (SD), respectively. Categorical data are presented as numbers (percentage) and were compared using the chi-square test. All tests were two-tailed, and a *p*-value of < 0.05 was considered statistically significant. The primary endpoints in this study were the occurrence of any colorectal neoplasia or advanced colorectal neoplasia in the screening colonoscopy. Advanced colorectal neoplasia was defined as previously described [12, 13]. The primary exposure was educational status, which was treated as a categorical fixed effect with higher education as the reference category. Multilevel logistic regression models were fitted with the year of inclusion as a random effect and the educational status as a categorical fixed effect (model-1). Further models included the covariables 10-year risk for cardiovascular disease and the family history of CRC (model-2) and age, sex, presence of metabolic syndrome, smoking status, family history of CRC, red meat consumption, and physical activity (model-3). Odds ratios (OR) and respective 95% confidence intervals (CI) were calculated for the binary dependent variables. All statistical analyses were performed using Stata/IC 17.

### Ethics statement

We performed the study and all procedures according to the principles of the Declaration of Helsinki. The local ethics committee for the province Salzburg approved the study protocol (approval no. 415-E/1262). Written informed consent was obtained from every participant.

## Results

The sample consisted of 2156 individuals with lower education, 2933 individuals with medium education, and 459 individuals with high education. The results showed that the mean age of individuals with lower education was 60 years (10), 57 years (9) for those with medium education, and 55 years (8) for those with high education (*p* < 0.001). With regard to sex, the results showed that 56% (1205/2156) of individuals with lower education were female, 43% (1249/2933) of individuals with medium education were female, and 42% (192/459) of individuals with high education were female (*p* < 0.001). Patients with lower education evidenced higher rates of cardiometabolic risk factors, and the mean 10-year risk of ASCVD (atherosclerotic cardiovascular disease) was 20% (16) for individuals with lower education, 18% (15) for those with medium education, and 14% (13) for those with high education (*p* < 0.001).

The cecal intubation was successful in ≥ 99% of individuals, independent of the educational status. With regard to any colorectal neoplasia, we found that 69% (1483/2156) of individuals with lower education, 67% (1967/2933) of individuals with medium education, and 68% (312/459) of individuals with high education had no neoplasia. Also, the mean amount of any neoplasia was similar between the groups: 0.54 (1.03) for individuals with lower education, 0.59 (1.70) for those with medium education, and 0.64 (2.20) for those with high education, with a *p*-value of 0.30 (Table 1).

**Table 1:** Baseline characteristics of patients with lower (GISCED 1 and 2), medium (GSISCED 3 and 4), and higher (GISCED 5 and 6) education. Most continuous variables were non-normally distributed. Continuous data are given as median ± inter-quartile range (IQR) and compared using Mann’s Whitney *U* test or mean ± standard deviation (SD) and compared using Student’s *T* test accordingly. Categorical data are given as numbers (percentage) and compared using the chi-square test. All tests were two-sided, and a *p*-value of < 0.05 was considered statistically significant

	Lower education	Medium education	High education	*p*-value
	*N* = 2156	*N* = 2933	*N* = 459	
Age (years)	60 (10)	57 (9)	55 (8)	< 0.001
Female sex (yes/no)	56% (1205)	43% (1,249)	42% (192)	< 0.001
Metabolic risk factors				
BMI (kg/m^2^)	28 (5)	27 (5)	26 (4)	< 0.001
Hypertension (yes/no)	63% (1,348)	55% (1,621)	43% (196)	< 0.001
Diabetes (yes/no)	17% (364)	16% (478)	14% (64)	0.30
Metabolic syndrome (yes/no)	73% (1581)	79% (2326)	75% (344)	< 0.001
ASCVD 10-year-risk (%)	20 (16)	18 (15)	14 (13)	< 0.001
Creatinine (mg/dL)	0.9 (0.2)	0.9 (0.2)	0.9 (0.2)	0.09
CRP (mg/dL)	0.4 (0.9)	0.3 (0.9)	0.2 (0.5)	< 0.001
Blood count				
Hemoglobin (g/dL)	14.5 (1.3)	14.7 (1.2)	14.6 (1.3)	< 0.001
MCV	86 (7)	88 (22)	87 (4)	0.62
Platelets (G/L	234 (63)	235 (56)	241 (59)	0.12
White blood count (G/L)	6.4 (2.1)	6.0 (1.6)	5.7 (1.5)	< 0.001
History				
Family history for CRC (yes/no)	11% (229)	11% (336)	14% (63)	0.15
Current smoking status				< 0.001
Never smoker	26% (377)	44% (1,183)	54% (243)	
Ex-smoker	45% (641)	36% (983)	30% (137)	
Active smoker	29% (418)	20% (541)	16% (72)	
Alcohol consumption				< 0.001
No alcohol	23% (448)	10% (289)	4% (16)	
< 2 drinks/day	56% (1,108)	83% (2,363)	92% (416)	
≥ 2 drinks/day	21% (413)	6% (182)	4% (19)	
Red meat (servings per week)	3 (2)	2 (2)	2 (1)	< 0.001
Physical activity per week				< 0.001
< 1 h	25% (444)	10% (231)	2% (8)	
1 to < 2 h	51% (926)	40% (901)	39% (132)	
2 to < 3 h	19% (343)	36% (821)	45% (154)	
≥ 3 h	5% (89)	13% (299)	14% (46)	

Patients with low (7%) educational status, patients with medium (8%) had similar, but patients with high (10%) educational status had higher rates of advanced colorectal neoplasia (Table 2). This difference was driven by higher rates of advanced colorectal neoplasia in the right-sided colon in patients with higher (8%) compared to low and medium (4% each) education (Table 2). The rates of advanced colorectal neoplasia in both the left sided colon and rectum did not differ significantly (Table 2).

**Table 2:** Endoscopic characteristics of patients with lower (GISCED 1 and 2), medium (GSISCED 3 and 4), and higher (GISCED 5 and 6) education. Most continuous variables were non-normally distributed. Continuous data are given as median ± inter-quartile range (IQR) and compared using Mann’s Whitney *U* test or mean ± standard deviation (SD) and compared using Student’s *T* test accordingly. Categorical data are given as numbers (percentage) and compared using the chi-square test. All tests were two-sided, and a *p*-value of < 0.05 was considered statistically significant

	Lower education	Medium education	High education	*p*-value
	*N* = 2156	*N* = 2933	*N* = 459	
Cecal intubation	99% (2125)	99% (2912)	100% (458)	0.010
Colorectal neoplasia				0.10
No neoplasia	69% (1483)	67% (1967)	68% (312)	
Not advanced neoplasia	24% (518)	25% (731)	22% (99)	
Advanced neoplasia	7% (155)	8% (235)	10% (48)	
Any neoplasia				
Mean amount of any neoplasia	0.54 (1.03)	0.59 (1.70)	0.64 (2.20)	0.30
Right-sided neoplasia	19% (414)	21% (609)	21% (98)	0.32
Left-sided neoplasia	15% (314)	14% (418)	15% (69)	0.89
Rectal neoplasia	6% (126)	5% (135)	2% (11)	0.004
Advanced neoplasia				
Right-sided advanced neoplasia	4% (85)	4% (127)	8% (35)	0.002
Left-sided advanced neoplasia	4% (84)	5% (141)	6% (27)	0.11
Rectal advanced neoplasia	2% (47)	2% (57)	2% (7)	0.63
Colorectal cancer	1% (19)	1% (27)	1% (3)	0.85

In the regression analyses, we observed an independent association between lower odds for advanced neoplasia and both medium and lower educational status compared to higher educational status (Table 3). This finding persisted after multivariable adjustment. However, this difference was entirely driven by the differences in the proximal colon (Table 3).

**Table 3:** We obtained odds ratios (OR) and respective 95%CI for the binary dependent variables using univariate and multivariable multilevel logistic regressions. The primary exposure was the educational status as categorical fixed effect with higher education as the reference category. Multilevel logistic regression models were fitted with the year of inclusion as a random effect and the educational status as a categorical fixed effect (model-1). Further models included additional covariates, the 10-year risk for cardiovascular disease, and the family history of CRC (model-2) and age, sex, presence of metabolic syndrome, smoking status, family history of CRC, red meat consumption, and physical activity (model-3). Odds ratios (OR) and respective 95% confidence intervals (CI) were calculated for the binary dependent variables

	Model-2			Model-3		
	Lower education	Medium education	Higher education	Lower education	Medium education	Higher education
Advanced neoplasia	0.62 (0.40–0.96); *p* = 0.03	0.65 (0.45–0.93); *p* = 0.02	Ref	0.51 (0.34–0.78); *p* = 0.002	0.66 (0.43–0.99); *p* = 0.046	Ref
Right-sided advanced neoplasia	0.42 (0.29–0.73); *p* = 0.001	0.41 (0.29–0.73); *p* = 0.001	Ref	0.28 (0.19–0.41); *p* < 0.001	0.42 (0.25–0.71); *p* = 0.001	Ref
Left-sided advanced neoplasia	0.63 (0.36–1.11); *p* = 0.11	0.69 (0.47–1.02); *p* = 0.06	Ref	0.51 (0.27–0.96); *p* = 0.04	0.68 (0.41–1.13); *p* = 0.14	Ref
Rectal advanced neoplasia	1.56 (0.46–5.30); *p* = 0.48	1.19 (0.46–3.09); *p* = 0.72	Ref	1.30 (0.36–4.62; p = 0.69	1.07 (0.39–2.91); *p* = 0.90	Ref

## Discussion

In this study, we evaluated a cohort of asymptomatic patients who underwent CRC screening colonoscopy by stratifying them based on their educational status. We aimed to understand the relationship between education and colorectal neoplasia prevalence and to identify any differences in baseline characteristics among patients in different educational classes. Our multivariable analysis corrected for these differences and found statistically significant differences in the frequency of advanced colorectal neoplasia, with only right-sided advanced colorectal neoplasia being different compared to left-sided or rectal advanced colorectal neoplasia.

There are prior studies that have associated higher educational status with higher rates of advanced colorectal neoplasia [16–18]. In a systematic review, Aarts et al. found that there are differences in Europe versus North America: Lower education is associated with lower risk of advanced colorectal neoplasia in Europe, but with higher risk in North America [8]. Lower physical activity in the context of their work [19] of patients with higher educational status but also increased consumption of meat and eggs [20] were discussed as possible pathophysiological explanations for the higher rates of advanced colorectal neoplasia in patients with higher educational status. Interestingly, in our study, the participants with lower education reported even less physical activity, whereas the amounts of red meat as main meal were different statistically, but this difference did not seem clinically relevant. There is also evidence that patients with lower education are less likely to participate in CRC screening [21, 22]. In line with these preliminary studies, the average age of patients with low education status was also higher in our study, indicating that these patients came for screening later in their life. Kuo et al. showed for the Taiwanese population that CRC mortality is higher in patients with lower educational status [7]. Because our study cohort included only those patients who participated in local opportunistic CRC screening, we cannot make a definitive statement about the overall population. On the other hand, in terms of baseline characteristics, our cohort reflects the average population. However, because our results suggest an effect of educational status on the distribution of risk for advanced colorectal neoplasia, congruent with the prior literature, specific strategies to increase the participation rates of socioeconomic classes appear worth considering [23].

Also consistent with prior literature, we found a site-specific effect of educational status on rates of advanced colorectal neoplasia [16, 18]. In both preliminary studies and our analysis, advanced colorectal neoplasia in the proximal colon in particular was more frequently diagnosed in patients with higher educational status. This underlines the validity of considerations that colorectal neoplasia in the proximal colon is influenced by lifestyle factors [24].

The current study has limitations to consider. Firstly, it is a retrospective investigation of prospectively collected data. Additionally, the questionnaires used to document physical activity, alcohol consumption, and dietary habits were not independently validated. This may have introduced measurement bias into the data. Further studies with larger sample sizes and independently validated data collection methods would be necessary to confirm our findings. Another limitation is that the study was performed in a single center in Austria, and the results may not be generalizable to other populations or healthcare systems. Additionally, we included patients that underwent CRC screening for the first time at our institution; therefore, we cannot exclude that some of the participants might have had a prior colonoscopy at another center. Naturally, we were only able to include patients in our analysis who underwent opportunistic screening, so it is important to consider the healthy screenee bias and the associated limitations in extrapolating our results to the general population. The sample size of the higher educational status group was small in comparison to the other groups, which may limit the ability to generalize the findings to a larger population. Also, the exact workup of the different pathophysiologies and tumorigenesis of colorectal neoplasia in the proximal versus distal colon versus rectum is beyond the scope of this study [25, 26]. We also cannot evaluate the possible significance of serrated versus conventional colorectal neoplasia with this study [27]. Despite these limitations, we believe that our analysis provides a valuable contribution to the literature due to the prospectively collected data, the size of the cohort, the use of the latest ISCED classification, and the large number of cardiometabolic and carcinogenic risk factors that we were able to adjust for in our relationship between educational status and colorectal neoplasia.

## Conclusion

Our study revealed that individuals with higher educational status have a higher prevalence of advanced colorectal neoplasia in comparison to those with medium and lower educational status. This association remained significant after controlling for other health parameters. However, it is worth noting that the rates of any neoplasia were consistent across all educational levels. Further investigation is necessary to comprehend the causes behind this difference, particularly in terms of the limited anatomical distribution of the observed difference. From a public health perspective, it is important to continue investigating why individuals with higher educational status have more advanced colorectal neoplasia in order to eliminate potential toxins or reduce associated lifestyle habits. From a clinical perspective, this information could be considered in clinical reasoning and patient counseling.

## Data Availability

Data are available upon reasonable request.
